# Predictors of peri-implant bone remodeling outcomes after the osteotome sinus floor elevation: a retrospective study

**DOI:** 10.1186/s12903-022-02592-6

**Published:** 2022-12-20

**Authors:** Xingxing Wang, Lijuan Sun, Lei Wang, Shaojie Shi, Sijia Zhang, Yingliang Song

**Affiliations:** 1 State Key Laboratory of Military Stomatology and National Clinical Research Center for Oral Diseases and Shaanxi Engineering Research Center for Dental Materials and Advanced Manufacture, Department of Oral Implants, School of Stomatology, The Fourth Military Medical University, Xi’an, 710032 Shaanxi People’s Republic of China; 2 State Key Laboratory of Military Stomatology and National Clinical Research Center for Oral Diseases and Shaanxi Engineering Research Center for Dental Materials and Advanced Manufacture, Department of Periodontology, School of Stomatology, The Fourth Military Medical University, Xi’an, 710032 Shaanxi People’s Republic of China; 3 Key Laboratory of Shaanxi Province for Craniofacial Precision Medicine Research, College of Stomatology, Xi’an Jiaotong University, Xi’an, 710004 Shaanxi People’s Republic of China; 4 920th Hospital of Joint Logistics Support Force, Kunming, People’s Republic of China

**Keywords:** Osteotome sinus floor elevation, Implant length, Residual bone height, Sinus membrane thickness, Vertical bone gain, Marginal bone loss

## Abstract

**Background:**

This study aimed to evaluate the radiographic outcomes of implants after osteotome sinus floor elevation (OSFE), and further identify the separate predictors for these radiographic outcomes.

**Methods:**

In this retrospective cohort study, a total of 187 implants were inserted into 138 patients using the OSFE technique. Seventy-four patients in the grafted group, and 64 patients in the non-grafted group completed this study. The vertical bone gain (VBG) and marginal bone loss (MBL) at 3 years following surgery were assessed as outcome variables. Based on extensive literature results, variables considered potential predictors of outcome variables included sex, age, tooth position, implant length, implant diameter, with or without grafting materials, residual bone height, sinus width, bone density, and sinus membrane thickness. Subsequently, the binary logistic regression analysis was applied with VBG and MBL as dependent variables, respectively. The receiver operating characteristic curve (ROC) with its area under the curve (AUC) was performed to further determine the predictive value of these predictors.

**Results:**

One hundred and six implants in grafted group and 81 implants in the non-grafted group were analyzed. The average VBG was 2.12 ± 1.94 mm for the grafted group and 0.44 ± 1.01 mm for the non-grafted group at 3 years (*P* < 0.05). The mean MBL was 1.54 ± 1.42 mm for the grafted group and 1.13 ± 1.69 mm for the non-grafted group at 3 years (*P* > 0.05). After the adjustment for confounders, logistic regression analysis demonstrated that implant length, grafting, residual bone height, and sinus membrane thickness were predictors of VBG. The odds ratio for VBG was 3.90, 4.04, 4.13 and 2.62, respectively. Furthermore, grafting exhibited the largest AUC at 0.80. While tooth position and implant length were predictors of MBL, the odds ratio for MBL was 3.27 and 7.85, respectively. Meanwhile, implant length exhibited the largest AUC at 0.72.

**Conclusions:**

OSFE with or without simultaneous grafting materials both showed predictable clinical outcomes. Additionally, the present study is the first quantitative and significant verification that VBG has a significant association with sinus membrane thickness, as well as residual bone height, implant length and grafting. Whereas tooth position and implant length are markedly associated with MBL.

## Introduction

Due to the pneumatization of the maxillary sinus and alveolar ridge resorption after tooth loss, inadequate alveolar bone height often challenges implant treatment in the atrophic posterior maxilla [1]. The osteotome sinus floor elevation (OSFE), first proposed by Tatum (1986) and subsequently improved by Summers (1994), is viewed as a well-documented technique for implant-supported rehabilitation [2]. Over the years, the use of grafting materials in OSFE has produced favorable clinical outcomes. Deproteinized bovine bone mineral has been frequently used for sinus elevation in that its good osteoconductive properties [3]. Studies have observed that the mean vertical bone gain (VBG) varied from 3.17 to 5.1 mm in the non-grafted group, whereas VBG varied from 1.7 to 4.1 mm in the grafted group, and more bone gain was gained with bone grafting [4, 5]. However, even in cases where the initial bone availability was 2 to 5 mm, spontaneous bone formation was also observed after the OSFE procedure without any grafting materials [6]. In addition, similar VBG and marginal bone loss (MBL) at every follow-up visit were reported in the grafted and non-grafted groups [7]. To date, the debate has increasingly arisen on the necessity of grafting materials. To the best of our knowledge, residual bone height is a pivotal criterion in the selection of sinus floor augmentation procedures. It was demonstrated, in a network meta-analysis comprising 20 studies and 1486 implants, that short implant insertion alone or OSFE with or without bone grafting has achieved better clinical results than the lateral window technique in terms of the residual bone height of > 5 mm [8]. Nevertheless, there is an argument regarding the association between clinical outcomes and residual bone height of < 5 mm. Studies have confirmed that the primary stability of simultaneous implants was provided by the cortical bone [9], which might be significantly reduced because of the bone contact only at the coronal area rather than full length [10], so the achievement of firm initial stability in a severely atrophic alveolar crest is of chief concern. During the OSFE technique, the bone compression and increased implant-bone contact area could in turn enhance the primary stability of implants and thereby promote osseointegration [11]. Moreover, recent studies also concluded that transalveolar sinus floor elevation with or without grafting material were both reliable therapeutic options for sinus augmentation with limited residual bone height [12, 13]. This was consistent with a randomized controlled study, where favorable clinical outcomes could be achieved following OSFE with a mean residual bone height of 2.4 ± 0.9 mm (range 0.9–4.0 mm) [14].

Due to the lack of periodontal ligament, osseointegrated oral implants are directly ankylosed to the surrounding bone, so the load transfer mechanisms are totally different from natural teeth. Maximum stress is concentrated on the implant neck area. The improper occlusion, implant designs and sites of placement could result in occlusal overload [15], which might activate biological bone resorption and subsequently comprise prosthesis longevity [16]. A three-dimensional finite element analysis has confirmed that implant designs and surgical sites could influence peri-implant load transmission [15]. In line with a previous study, increased implant length could reduce stress at the implant-abutment interface during the osseointegration phase, but no specific correlation was found regarding the influence of implant diameter [17].

It appeared that bone density played a determinant role in achieving primary stability after sinus floor elevation with a residual bone height of 2–6 mm [10]. Recently, with the popularity of cone beam computed tomography (CBCT) in implant treatment, bone density could be assessed precisely with a value (Hounsfield units: HU). Specifically, the bone density classification based on computed tomography values was categorized as follows [18, 19]: D4, 150–350 HU; D3, 350–850 HU; D2, 850–1250 HU; D1, > 1250 HU. In the posterior maxilla region, more than 80% of bone quality was classified as D3 (32%) or D4 (50%). Hence, instead of correlating the implant success rate with tooth position alone, bone quality could be also viewed as a crucial predictor [20].

Apart from the above-mentioned factors, anatomic variations such as the sinus width and Schneiderian membrane thickness might be of utmost significance to the new bone formation [21, 22]. During the OSFE surgery, the wider sinus might hinder the exposure of lateral and medial sinus walls, thereby create a low regenerative potential environment and influence new bone formation [23]. But there is a lack of unified classification criteria for sinus width. Currently, the osteogenic potential of human maxillary Schneiderian sinus membrane has been confirmed on account of the isolation of mesenchymal osteoprogenitor cells in vitro study [24]. With the OSFE surgery, the elevated membrane could also protect the stability of a blood clot and promote the osteogenesis process [25]. The normal sinus membrane thickness ranged from 0.8 mm to 1.99 mm [26], and 2 mm was counted as a threshold for pathological membrane thickening [27]. A recent review reported that sinus membrane thickening had no correlation with complications and implant survival rate [28]. Nevertheless, because of the high heterogeneity among studies, no clear conclusions could be made on the correlation between mucosal thickening and implant survival.

Hence, the purpose of this retrospective study was to evaluate peri-implant clinical outcomes following the OSFE approach with or without grafting. A further purpose was to explore the separate predictors for these clinical outcomes.

## Methods

### Study design

One hundred and thirty-eight consecutive patients were eligible for this retrospective cohort study. The OSFE surgery was performed from January 2015 to December 2017 at the Department of Oral Implants, The Affiliated Stomatology Hospital, Fourth Military Medical University, China. The follow-up period was 3 years. For publishing purposes, this retrospective cohort study was approved by the Ethnic Committee of School of Stomatology, the Fourth Military Medical University (Approval No. IRB-REV-2022082) and was in strict conformity with Helsinki Declaration. All patients were acquainted with the complete treatment scheme and signed informed consent before surgery. The study protocol was conducted following the Strengthening the Reporting of Observational Studies in Epidemiology (STROBE) statements.

### Patient selection

Patients were recruited by the following inclusion criteria: (1) age > 18 years and in good oral hygiene; (2) partial edentulism in the maxillary posterior area for at least 3 months from tooth loss; (3) available residual bone height ranging from 0.96 to 8.5 mm; (4) without rhinitis or sinusitis; (5)without previous history of sinus operation or grafting at the implant site; (6) complete case records and imaging data.

Exclusion criteria were as follows: (1) uncontrolled periodontal lesions or other oral disorders during follow-up visits; (2) systematic illness or under any medication influencing bone metabolism after the surgery; (3) head and neck radiation treatment during follow-up periods; (4) heavy smokers (≥ 20 cigarettes per day) [29]; (5) technical artifacts influencing data assessment; (6) partial sinus visibility in CBCT scans; (7) membrane perforation during the OSFE procedure; (8) bone excision for oncological treatment before or after the surgery.

### Surgical procedure

A pre-surgical CBCT was performed to critically appraise the residual bone height and anatomical structure of the maxillary sinus. All OSFE techniques were carried out by the corresponding author under local anesthesia and strictly sterile conditions. After a mid-crestal incision, a full-thickness mucoperiosteal flap was raised to visualize the alveolar crest. Then the implant socket was prepared to a depth of 0.5–1 mm below the sinus floor, as measured on pre-operative CBCT radiographs, and the maxillary sinus floor was elevated using a set of osteotomes with different diameters. After examining the integrity of the Schneiderian membrane utilizing the Valsalva maneuver, a combination of deproteinized bovine bone minerals (Bio-Oss, large particles; Geistlich, Wolhusen, Switzerland) and autogenous bone chips were mixed to fill the antrum at this stage. Finally, the Straumann implant (Straumann AG, Waldenburg, Switzerland) or Nobel Active implant (Nobel Biocare, Zürich, Switzerland) was inserted and the flap was sutured. During each step of the OSFE operation, the sinus membrane perforation was evaluated by the Valsalva maneuver. For the non-grafted group, no grafting materials were used before implant insertion. Standardized digital panoramic radiography was taken immediately to determine the gain in vertical bone height and implant positions. After the surgery, nasal decongestants, antibiotics, and mouth rinsing were prescribed for 1 week with their proper dosage. An analgesic was used to control post-surgical pain when needed. Sutures were removed 10 days after surgery.

### Radiographic evaluation

The radiographic evaluation was conducted on CBCT and standardized digital panoramic images. All images were taken by professional radiologists from the Department of Imaging, and all data were kindly provided by the Department of Information in our hospital. For each patient, a CBCT scan was performed before surgery to assess the residual bone height, bone density, sinus width, and sinus membrane thickness. Subsequently, digital panoramic radiographs (immediately after the operation) and CBCT radiographs (3 years post-surgery) were taken to measure outcome variables. Data were measured by the software (Mimics 20.0, Materialise Co., Ltd, Leuven, Belgium). The precision of the measuring system was 0.01 mm. To account for any panoramic distortion, radiographic measurements were calibrated by the known size of implants. All data obtained from panoramic images were adjusted for a coefficient derived from the ratio: “true implant length/radiographic implant length”. All linear measurements were conducted by two trained examiners.

**Fig. 1:**
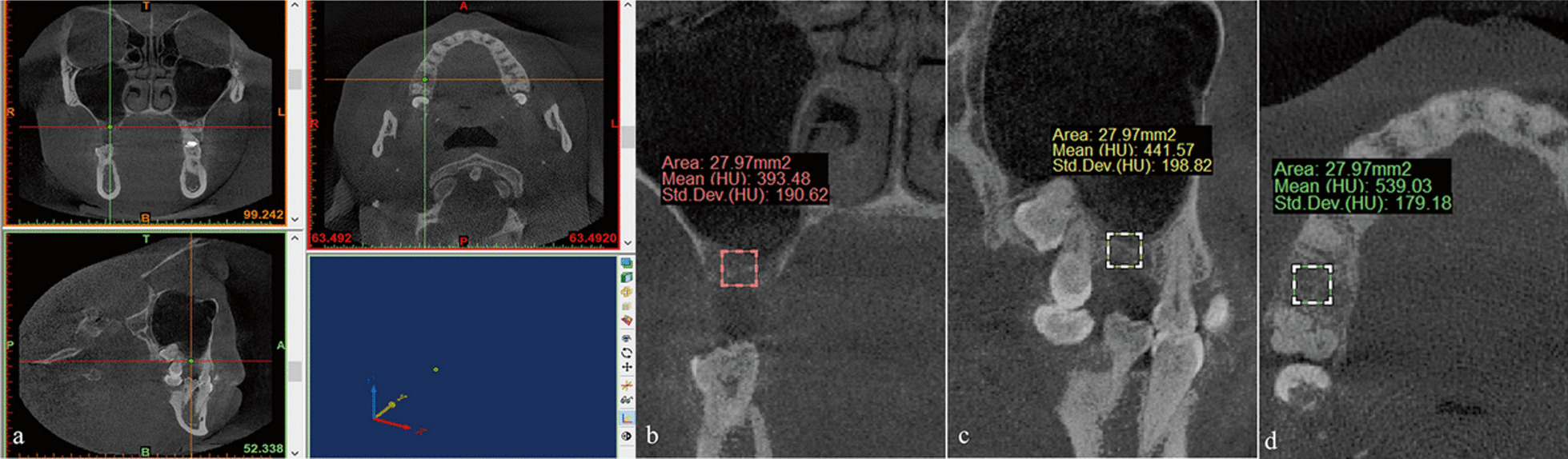
Measurement of bone density. Simulated images in the posterior maxilla area were analyzed on preoperative CBCT. **a** Measurement of bone density in three sections; **b** a coronal section; **c** a sagittal section; **d** a transverse section

**Fig. 2:**
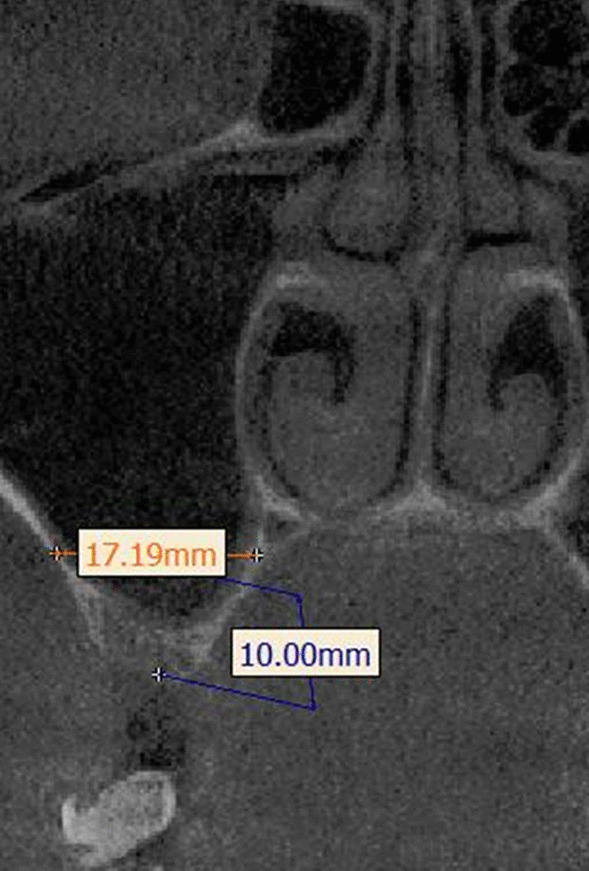
Measurement of sinus width. Sinus width was measured by the distance from the buccal to palatal walls at a height of 10 mm above the alveolar crest

CBCT data were acquired before OSFE surgery to appraise the following indicators:

1. Residual bone height: the distance from the cortical bone line to the sinus floor cortical bone line.
2. Bone density: the average grayscale value of three different sections (coronal section, transverse section, and sagittal section) measured in a prefixed area (Fig. 1).
3. Sinus width: the distance from the buccal to palatal wall measured at a height of 10 mm above the alveolar crest [30] (Fig. 2).
4. Sinus membrane thickness: the mean value was evaluated at nine distinct locations [27].

Subsequently, the parameters of outcome variables were measured as follows:

1. Radiographic implant length: the distance from the implant shoulder to implant apex measured on the panoramic radiograph.
2. Vertical bone height: the mean distance from the coronal bone-to-implant contact area to the newly formed sinus floor at the mesial and distal side of the implant.
3. Crestal bone level: the average distance from the implant apex to the coronal bone-to-implant area measured at the mesial and distal side of the implant.

Additionally, the following parameters were calculated:

1. Vertical bone gain (VBG): was calculated by subtracting the residual bone height from vertical bone height 3 years after surgery.
2. marginal bone loss (MBL): was calculated by subtracting the crestal bone level at 3 years from that immediately after the surgery.

### Study variables

In the present study, the outcome variables were VBG and MBL at 3 years after surgery, separately. They could be either a positive number, zero or a negative number. Other potential predictive variables that may be associated with the outcome variables included sex, age, tooth position, implant length, implant diameter, with or without grafting materials, residual bone height, bone density, sinus width and sinus membrane thickness. Continuous variables were converted into binary categorical variables within some specific criteria, and the details were as follows:

1. Sinus membrane thickness: was categorized as either normal (≤ 2 mm) or pathological (> 2 mm) [28].
2. Sinus width: was divided as either narrow sinus (≤ 12.1 mm) or wide sinus (> 12.1 mm) [31].
3. Bone density: was classified as either D3 and D4 (≤ 850 HU) or D1 and D2 (> 850 HU) [19].

### Statistical analysis

All data were analyzed via SPSS Statistics for Windows software (version 26.0, SPSS Inc., Chicago, IL, USA). Normally distributed quantitative variables were summarized as means ± standard deviations and analyzed by the t-test. While abnormally distributed quantitative variables were presented as medians and interquartile ranges (IQRs), and compared with the Mann–Whitney U test. The categorical variables were expressed as frequencies and percentages, and analyzed by the Pearson's chi-squared test. To analyze the predictors of VBG and MBL, the binary logistic regression analysis was performed and odds ratio (OR) values with the corresponding 95% confidence interval (CI) were calculated, respectively. Thus, receiver operating characteristic (ROC) curves were plotted based on the significant predictive variables with *P* < 0.05 in the logistic regression analysis. Furthermore, an area under the curve (AUC) was calculated to evaluate the capability of different variables in predicting VBG and MBL, separately. All tests were conducted using a two-sided test, and the level of statistical significance was set at *P* < 0.05.

## Results

### Baseline characteristics

From January 2015 to December 2017, a total of 187 implants in 138 patients (grafted group: 74, non-grafted group: 64) were enrolled in this clinical study. The median age was 48 years (IQR 41–52.25 years) for the grafted group and 48.5 years (IQR 43.25–54 years) for the non-grafted group (*P* = 0.36). During the 3-year follow-up, all implants successfully attained uneventful osseointegration and achieved functional loading with permanent prostheses. Meanwhile, the implant survival rate was 100% in both groups. Table 1 summarized the implant and patient information between the two groups. The mean residual bone height for the grafted group (5.26 ± 1.73 mm) was significantly lower in comparison with the non-grafted group (6.47 ± 1.02 mm), whereas other variables showed no statistically significant difference between the two groups.

**Table 1:** Comparison of variables between the grafted and non-grafted groups

Variables	Implant distribution		*P* value
	Grafted (n = 106)	Non-grafted (n = 81)	
Sex, n (%)			0.44^a^
Male	43 (58.11)	33 (51.56)	
Female	31 (41.89)	31 (48.44)	
Age (years), median (IQR)	48 (41, 52.25)	48.50 (43.25, 54.00)	0.36^b^
Tooth position, n (%)			0.47^a^
Premolar	19 (17.92)	18 (22.22)	
Molar	87 (82.08)	63 (77.78)	
Implant system, n (%)			0.75^a^
Straumann AG	87 (82.08)	65 (80.25)	
Nobel active	19 (17.92)	16 (19.75)	
Implant diameter (mm), n (%)			0.55^a^
≤ 4.1	75 (70.75)	54 (66.67)	
> 4.1	31 (29.25)	27 (33.33)	
Implant length (mm), n (%)			0.11^a^
≤ 8	23 (21.70)	26 (32.10)	
> 8	83 (78.30)	55 (67.90)	
SW (mm), mean ± SD	13.42 ± 2.99	14.07 ± 3.68	0.20^c^
BD (HU), median (IQR)	550.04 (476.48, 635.08)	563.14 (457.40, 663.98)	0.66^b^
SMT (mm), Median (IQR)	1.84 (1.72, 2.10)	1.95 (1.72, 2.49)	0.26^b^
RBH (mm), mean ± SD	5.26 ± 1.73	6.47 ± 1.02	**0.00**^c^

*SW* sinus width, *BD* bone density, *SMT* sinus membrane thickness, *RBH* residual bone height, *IQR* interquartile range, *SD* standard deviation

^a^Chi-squared test; ^b^Mann–Whitney U test; ^c^t-test

### Radiographic assessment

The VBG and MBL at 3 years after surgery are listed in Table 2. The mean VBG was 2.12 ± 1.94 mm (grafted group) and 0.44 ± 1.01 mm (non-grafted group), respectively, and there was a significant increase in VBG for the grafted group (*P* = 0.00). The average MBL was 1.54 ± 1.42 mm for the grafted group and 1.13 ± 1.69 mm for the non-grafted group, and no significant difference was found between the two groups (*P* = 0.07).

**Table 2:** VBG and MBL at 3 years after surgery (mean ± SD, mm)

Groups	n	VBG	MBL
Grafted	106	2.12 ± 1.94	1.54 ± 1.42
Non-grafted	81	0.44 ± 1.01	1.13 ± 1.69
*P* value		**0.00**	0.07

*VBG* vertical bone gain, *MBL* marginal bone loss, *SD* standard deviation

### Variables related to the VBG

**Fig. 3:**
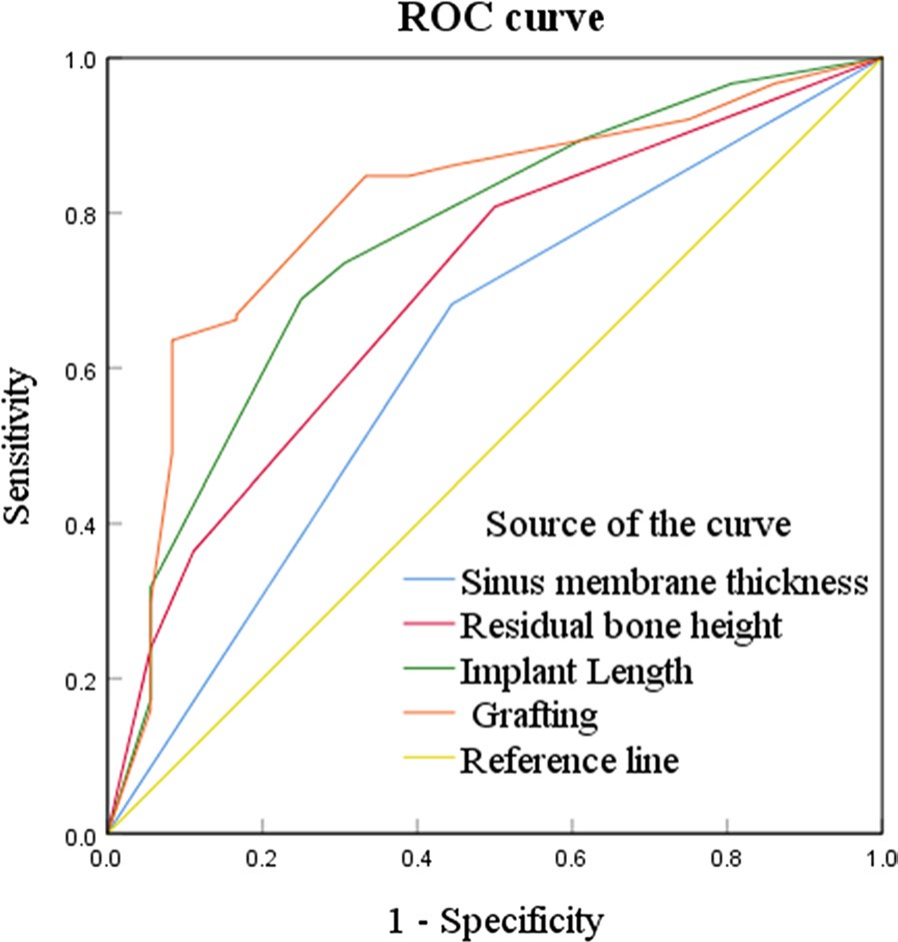
The ROC curve of VBG was established. *ROC curve* receiver operating characteristic curve, *VBG* vertical bone height

A binary logistic regression analysis was performed on the predictors of VBG based on the previous literature results. VBG 3-years post-surgery was used as a dependent variable, and adjusting for sex, age, tooth position, implant length, implant diameter, with or without grafting, residual bone height, sinus width, bone density, and sinus membrane thickness as independent variables for VBG. In binary logistic regression analysis, grafting (OR 4.04, 95% CI 1.55–10.50), implant length (OR 3.90, 95% CI 1.42–10.66), residual bone height (OR 4.13, 95% CI 1.11–15.36), sinus membrane thickness (OR 2.62, 95% CI 1.09–6.28) emerged as independent predictors for VBG, with *P* = 0.01, *P* = 0.004, *P* = 0.04, and *P* = 0.03, respectively (Table 3). Additionally, in ROC curve analysis, the AUC for grafting, implant length, residual bone height and sinus membrane thickness were 0.80, 0.76, 0.70 and 0.62, separately (Table 4; Fig. 3).

**Table 3:** Logistic regression analysis for VBG with predictors

Variables	β	SE	OR	95% CI	*P* value
Grafting	1.395	0.49	4.04	(1.55, 10.50)	**0.004**
SMT (mm)	0.96	0.45	2.62	(1.09, 6.28)	**0.03**
RBH (mm)	1.42	0.67	4.13	(1.11, 15.36)	**0.04**
Implant length (mm)	1.36	0.51	3.90	(1.42, 10.66)	**0.01**

*VBG* vertical bone gain, *SMT* sinus membrane thickness, *RBH* residual bone height, *β* regression coefficient, *SE* standard error, *OR* odds ratio, *CI* confidence interval

**Table 4:** ROC analysis for VBG with predictors

Variables	AUC	SE	Asymptotic Sig	95% CI
Grafting	0.80	0.04	0.00	(0.72, 0.88)
SMT (mm)	0.62	0.05	0.03	(0.52, 0.72)
RBH (mm)	0.70	0.05	0.00	(0.61, 0.80)
Implant length (mm)	0.76	0.05	0.00	(0.68, 0.85)

*ROC* receiver operating characteristic curve, *VBG* vertical bone gain, *SMT* sinus membrane thickness, *RBH* residual bone height, *AUC* area under the curve, *SE* Standard error, *CI* confidence interval

### Variables related to the MBL

**Fig. 4:**
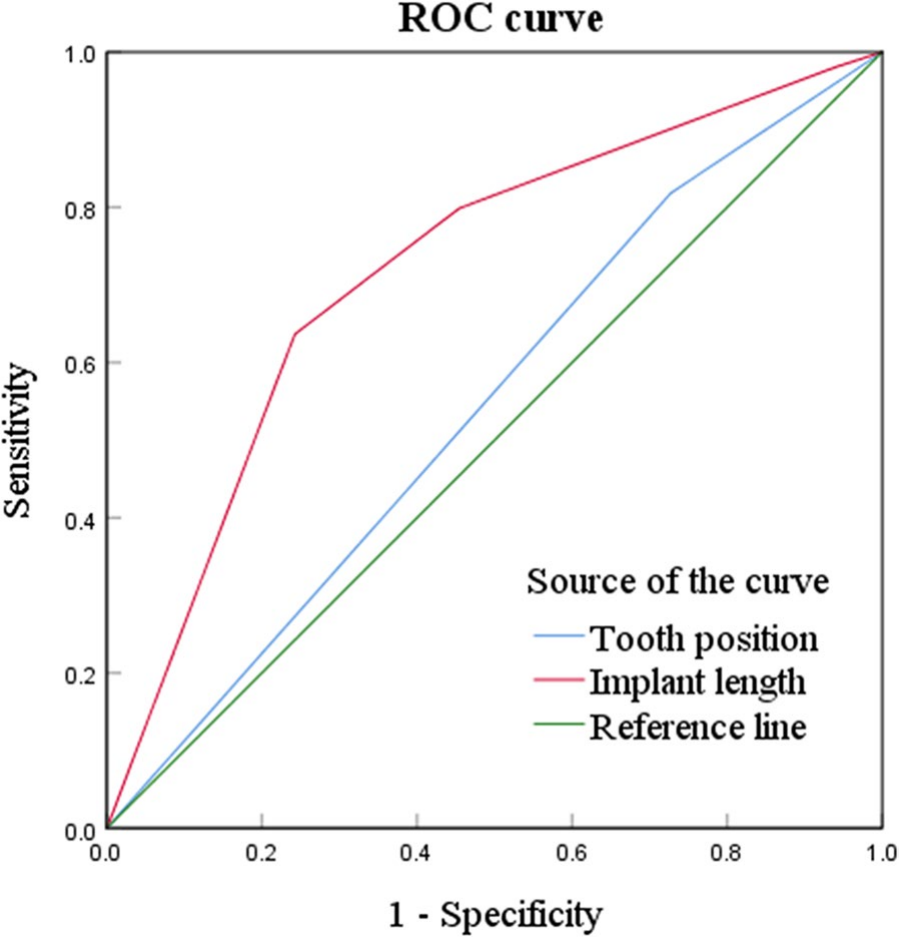
The ROC curve of MBL was established. *ROC curve* receiver operating characteristic curve, *MBL* marginal bone loss

The binary logistic regression analysis was applied to detect predictive factors of MBL. MBL 3-years post-surgery was used as the dependent variable, and adjusting for sex, age, tooth position, implant length, implant diameter, residual bone height, and bone density as independent variables for MBL. The results demonstrated that tooth position (OR 3.27, 95% CI 1.18–9.03) and implant length (OR 7.85, 95% CI 3.04–20.29) were independent predictors for MBL, with *P* = 0.02 and *P* = 0.00, respectively (Table 5). In ROC curve analysis, the AUC for tooth position and implant length were 0.56 and 0.72, separately (Table 6; Fig. 4).

**Table 5:** Logistic regression analysis for MBL with predictors

Variables	β	SE	OR	95% CI	*P* value
Tooth position	1.18	0.52	3.27	(1.18, 9.03)	**0.02**
Implant length (mm)	2.06	0.48	7.85	(3.04, 20.29)	**0.00**

*MBL* marginal bone loss, *β* regression coefficient, *SE* standard error, *OR* odds ratio, *CI* confidence interval

**Table 6:** ROC analysis for MBL with predictors

Variables	AUC	SE	Asymptotic Sig	95% CI
Tooth position	0.56	0.06	0.41	(0.43, 0.66)
Implant length (mm)	0.72	0.05	0.00	(0.62, 0.82)

*ROC* receiver operating characteristic curve, *MBL* marginal bone loss, *AUC* area under the curve, *SE* standard error, *CI* confidence interval

## Discussion

This retrospective cohort trial aimed at evaluating the peri-implant clinical outcomes between different treatment groups, and further analyzing the separate predictors of radiographic outcome indicators after the OSFE technique.

VBG is one of the significant indicators to evaluate the clinical outcome of the OSFE procedure. In this study, the average VBG for the grafted and non-grafted groups at 3 years was 2.12 ± 1.94 mm and 0.44 ± 1.01 mm, respectively, and a statistically significant difference was found between the two groups (*P* < 0.05). This was in line with a recent meta-analysis, which showed a significantly lower VBG with graft less sinus lift [32]. In addition, grafting (OR 4.04) was found to be positively correlated with VBG in this paper. When the available bone height is inadequate in an atrophic posterior maxilla, clinicians tend to utilize grafting materials to create adequate space for subsequent implant insertion. Under the augmented sinus membrane, the simultaneous placement of deproteinized bovine bone mineral acts as an osteoconductive scaffold that allows cell migration and subsequent de novo bone formation [33]. Thus the endo-sinus bone gain was significantly higher, compared with the OSFE technique without simultaneous grafting. We also found the 3-year survival rate of implants in both groups was 100%, which agreed with a recent meta-analysis that there was no significant difference in the long-term survival rate of implants after the OSFE surgery [34]. These favorable results may be explained as follows: In the posterior maxillae, the implant may offer enough osseointegrated bone-to-implant area to withstand the masticatory force, which is essential to ensure the long-term clinical outcomes of implant rehabilitation [35]. From the biomechanical perspective, the occlusal force would mainly concentrate on the crestal bone. Hence, providing that osseointegration meets the loading requirement, new bone formation after the sinus membrane elevation perhaps occurs.

To the best of our knowledge, this is the first clinical evidence that sinus membrane thickness (OR 2.62) had a significant correlation with VBG. During the sinus lift approach, the key lay in the full detachment of the Schneiderian membrane from the bony sinus floor, which was an essential precondition for bone regeneration underneath the maxillary sinus [36]. Meanwhile, the membrane integrity was crucial to the subsequent bone reformation process [37]. It should be noted that, nevertheless, the elevated operation and implant insertion proceeded blindly, which increased the interoperative perforation risk of sinus membrane. Janner and his colleagues reported that the thickness of the Schneiderian membrane ranged from 0.16 mm to 34.14 mm, and there existed great interindividual variability regarding the membrane thickness [26]. It was reported that the sinus membrane thickness ranging from 1 to 2 mm carried the lowest perforation risk [38], which may be explained by the higher load limits of the membrane. But there is controversy regarding the association between membrane perforation and implant failure rate. A previous systematic review including 7358 implants indicated that membrane perforation in the sinus augmented procedure could increase the risk of implant failure [39]. Additionally, a recent multicenter study of 430 implants, also confirmed that the sinus membrane perforation [OR 4.21; 95% CI (1.10–16.05); *P* = 0.035] had a statistically significant effect on early implant failure [40]. This could be explained as follows: the membrane perforation may result in local inflammation, influence the remolding of grafting material, reduce bone formation and ultimate implant loss. To a certain extent, the debate could be interpreted by the different sizes of membrane perforation among studies, which was a significant confounding factor. Owing to the smaller perforation size may maintain the integrity of the lifting procedure, the implant’s clinical effectiveness was not modulated by membrane perforation.

The amount of new bone formation after the sinus lift procedure is crucial to the outcome of implants [41]. In the present study, residual bone height (OR 4.13) and implant length (OR 3.90) both had a significant correlation with VBG. This result agreed with a recent clinical study, where the higher difference between implant length and residual bone height was, the more bone height was gained [42]. It was also consistent with other studies where the length of the implants protruding into the maxillary sinus and residual bone height both were important factors in new bone formation [43, 44]. Conversely, the residual bone height was found to have no significant association with the amount of new bone formation [45], this might be on account of its indirect impact on the bone remolding. With the sinus lift technique, the implant fixture acted as tent poles to stabilize the blood clot in close contiguity with important structures. In the case of grafting materials placed under the sinus, the blood clot and grafting materials both performed their role in providing the space for new bone formation. However, the space finally was sustained by the implant and bone grafting materials after the remolding, in that the blood clot and partial bone substitutes were resorbed over time [46]. Since the grafting and VBG were significantly correlated in this paper, to a certain extent, the lower residual bone height, the higher the membrane was elevated, with grafting materials placed into the sinus floor, and the more bone would be created.

Sinus floor elevation is a well-documented method to achieve implant prosthesis in an atrophic maxilla. The angiogenesis and osteoblasts play a crucial role in endo-sinus new bone formation [47]. By virtue of vascular supply and osteoprogenitor cells involving new bone formation mainly come from the surrounding bony walls [48], the wider sinus may need more bone substitutes and longer healing time to achieve an acceptable amount of newly formed bone [49–51]. The intro-sinus bone formation has been verified to be more in narrow sinuses than in wider sinuses [52, 53]. Meanwhile, based on the logistic regression analysis, a significant correlation was found between larger sinus cavities [OR 8.50; 95 CI (1.02–70.42); *P* = 0.047] and early implant failure [40]. However, this study did not find any association between sinus width and VBG, which could be partly explained by methodological discrepancies among studies. In the previous literature, there are diverse categorizations concerning sinus width, both the bone crest and sinus floor level could be viewed as a standardized reference to assess sinus width [54], but this paper adopted a new classification system with good visualization and feasibility [30]. Furthermore, different surgical approaches for sinus floor elevation, different bone grafting materials, different healing durations, different radiographical examinations and different biopsy techniques all could be considered confounding variables, which may elucidate differences among studies outcomes.

Implant primary stability is a prerequisite to successful osseointegration. During the bone healing period, insufficient initial stability may enhance micromotion at the bone-implant surface, endanger osseointegration and thereby induce the encapsulation of fibrous tissue and hypertrophy of the trabecular bone [55]. A previous clinical study demonstrated that higher bone density values (Hounsfield units) correlated with higher initial stability measured by insertion torque and resonance frequency analysis [56]. Nevertheless, there was no correlation between VBG and bone density in our study. One possible explanation for this result is that the implant primary stability was affected by many factors, such as implant design, implant surface morphology, surgical approach and bone quality [57]. Consequently, further prospective clinical trials with larger sample sizes are needed to corroborate the correlation between bone density and VBG.

MBL is one of the most frequently reported indicators to assess the long-term clinical outcome of implants. No significant difference was found between the different treatment groups. This result was consistent with a previous systematic review [58]. Furthermore, we also found that MBL had a significant correlation with tooth position (OR 3.27), as well as implant length (OR 7.85). This result was confirmed by a 6-year study, where the MBL was higher in the maxillary posterior region [59]. For osseointegrated dental implants, implants placed in molar regions usually withstand a greater occlusal force than the premolar areas. As the occlusal force during functional load is mainly distributed in the implant neck area, the occlusal overload in molar areas could easily lead to the occurrence of MBL.

In addition, studies observed greater MBL with longer implants after the sinus elevation approach [60, 61], which was in line with our results. In an atrophic maxillary region, longer implants had biomechanical advantages to reduce stress in the implant neck area, but as the implant’s tilting degree increased, stress values at the implant neck area increased somewhat, and the impact of tilting degree was more dominant than the implant length [62]. In addition, longer implants might lead to early failure due to the absence of initial stability. It is important to note, however, that the implant length and implant diameter did not show any statistically significant influence on MBL [63]. These findings may be related to the following facts: where peri-implant MBL was influenced by many ingredients, for example, crown/implant ratio, excessive cantilever length, biological factors, systematic factors, different grafting materials, surgical preparation and implant geometry [63, 64].

Nevertheless, the results of this retrospective study should be interpreted with caution due to the follow-up time. Additionally, a digital panoramic radiograph after surgery was taken to calculate the outcome variables, which may be regarded as a source of bias. So future studies with larger sample sizes, longer follow-up periods and CBCT evaluation at each follow-up visit remain essential.

## Conclusion

Within the limitations of this retrospective study, some conclusions can be drawn as follows:

1. In the posterior maxilla, OSFE with or without grafting materials both resulted in predictable clinical outcomes, and more bone formation was observed with grafting materials.
2. The present study is the first quantitative and significant verification that sinus membrane thickness has a positive correlation with VBG.
3. No significant association was observed between sinus width, bone density and VBG, respectively.
4. Residual bone height and implant length had a significant correlation with VBG, separately.
5. Tooth position and implant length had a significant correlation with MBL, respectively.

## Data Availability

The datasets used and analyzed during the current study are available from the corresponding author upon reasonable request.

## Abbreviations

OSFE: Osteotome sinus floor elevation
VBG: Vertical bone gain
MBL: Marginal bone loss
HU: Hounsfield units
CBCT: Cone beam computed tomography
IQRs: Medians and interquartile ranges
OR: Odds ratio
CI: Confidence interval
ROC: Receiver operating characteristic curve
AUC: Area under the curve
